# The heme-regulated inhibitor kinase Hri1 is activated in response to aminolevulinic acid deficiency in *Schizosaccharomyces pombe*

**DOI:** 10.1371/journal.pgen.1011797

**Published:** 2025-07-16

**Authors:** Samuel Plante, Ariane Brault, Mariano Avino, Hajer Sakouhi, Florie Lo Ying Ping, Tobias Vahsen, Simon Labbé

**Affiliations:** Département de Biochimie et de Génomique Fonctionnelle, Faculté de médecine et des sciences de la santé, Université de Sherbrooke, Sherbrooke, Canada; The Francis Crick Institute, UNITED KINGDOM OF GREAT BRITAIN AND NORTHERN IRELAND

## Abstract

A key mechanism for regulating the initiation of protein synthesis in response to various stresses involves the phosphorylation of the α subunit of eukaryotic initiation factor 2 (eIF2α). *Schizosaccharomyces pombe* possesses three distinct eIF2α kinases: Hri1, Hri2, and Gcn2. Using a strain that is unable to synthesize heme *de novo* (*hem1*Δ), global transcriptome analysis reveals that among the genes encoding these kinases, *hri1*^*+*^ is the most strongly induced under δ-aminolevulinate (ALA)-limiting conditions. The induction of *hri1*^*+*^ consistently correlates with increased eIF2α phosphorylation and a reduction in global protein translation in ALA-starved *hem1*Δ cells. In contrast, *hem1*Δ cells lacking *hri1*^*+*^ (*hri1*Δ) exhibit poor eIF2α phosphorylation under the same stress conditions. When ALA-starved *hem1*Δ *hri1*Δ cells are subsequently transferred to a medium supplemented with exogenous hemin, they exhibit impaired growth compared to ALA-starved *hem1*Δ cells expressing the endogenous *hri1*^*+*^ allele or *hem1*Δ *hri1*Δ* hri2*Δ* gcn2*Δ cells expressing functional *hri1*^*+*^ and *hri1*^*+*^*-GFP* alleles. Consistent with its role as a heme-sensing eIF2α kinase, further analysis by absorbance spectroscopy demonstrates that Hri1 binds to hemin, with an equilibrium dissociation constant (*K*_*D*_) of 0.11 µM. In contrast, a truncated form of Hri1 (from residues 1–185) fails to interact with hemin. Taken together, these findings provide the first report of a fungal eIF2α kinase being activated in response to stress directly linked to a defect in heme homeostasis.

## Author summary

Yeasts encounter various environmental stresses to which they must adapt for survival. Among the strategies they have developed, one involves restricting global protein translation to reprogram their machinery for the synthesis of protective proteins against specific stresses. Fission yeast possesses three protein kinases capable of phosphorylating the translation factor eIF2α, thereby inhibiting global translation. In this study, Hri1 is specifically induced in response to aminolevulinate (ALA) deficiency, a heme precursor. Under this stress condition, eIF2α is phosphorylated in an Hri1-dependent manner. ALA-starved cells lacking Hri1 exhibit impaired growth when transferred to a medium supplemented with exogenous hemin. In contrast, cells deleted for all three eIF2α kinase genes but expressing Hri1 are able to restore hemin-dependent growth under these conditions. Biochemical analysis reveals that Hri1 has a hemin-binding affinity one order of magnitude greater than that of Hri2, a related eIF2α kinase. These findings establish Hri1 as the first fungal eIF2α kinase activated under heme-deficient conditions to reduce global protein synthesis, thereby facilitating adaptation to this stress.

## Introduction

Heme is an essential molecule composed of a protoporphyrin ring, with an iron atom coordinated at its center. A key property of heme is the ability of its bound iron to reversibly switch between oxidation states (Fe^2+^ to Fe^3+^, and vice versa). This redox-active nature of iron makes heme a critical cofactor for several enzymes that mediate electron transfer and oxidation-reduction reactions in cells [1–5]. Beyond its role as a cofactor, heme also functions as a signaling molecule by binding to or dissociating from regulatory proteins, including transcription factors and stress kinases [6,7]. One such example is the heme-regulated inhibitor (HRI) protein, which acts as a heme-sensing kinase in mammalian cells [8,9]. Under heme-replete conditions, HRI is inhibited through direct association with heme. However, when cellular heme levels drop, heme dissociates from HRI, triggering its activation. This activation leads to HRI autophosphorylation and the subsequent phosphorylation of the α subunit of eukaryotic initiation factor 2 (eIF2α) [10–15]. Phosphorylation of eIF2α at a highly conserved Ser residue (Ser51 in mammals) inhibits the guanine nucleotide exchange activity of the translation initiation factor eIF2B. As a result, the formation of the active eIF2-GTP complex, which is required for recruiting methionyl-initiator tRNA to the translational machinery, is dramatically reduced. This suppression of eIF2B activity effectively prevents new protein synthesis [16,17]. In mammals, HRI activation in response to heme deficiency plays a crucial role in erythroid cells by inhibiting globin translation, thereby preventing the accumulation of nonfunctional, heme-free globin chains [8,18].

The regulation of translation initiation through eIF2α phosphorylation is a key component of a broader, evolutionarily conserved pathway known as the integrated stress response (ISR) [19–23]. In response to various environmental and cellular stresses, the ISR downregulates global mRNA translation while selectively promotes the translation of a limited set of transcripts. These transcripts encode proteins that help mitigate stress or support cell survival under specific conditions.

In mammals, in addition to HRI, three other kinases inhibit the initiation of protein translation by phosphorylating eIF2α at Ser51. These kinases are PKR, PERK, and GCN2 [23,24]. Although all four proteins share conserved kinase domains, each has distinct regulatory domains that enable them to respond to different types of stress. PKR is activated by interferon and the presence of double-stranded RNA during viral infection [25]. PERK activation depends on the accumulation of unfolded proteins in the secretory pathway [26,27]. GCN2 is induced by amino acid or serum starvation, exposure to UV radiation, or RNA viruses [28–30].

In *Schizosaccharomyces pombe*, environmental and cellular stresses that activate the ISR are sensed by three eIF2α kinases [31]. Hri1 and Hri2 are homologous to mammalian HRI, whereas Gcn2 is considered an ortholog of *Saccharomyces cerevisiae* Gcn2, the sole eIF2α kinase in this yeast [32]. Unlike Hri1 and Hri2, Gcn2 lacks a putative heme-binding domain. A distinctive feature of the ISR in *S. pombe* is that phosphorylation of the α subunit of eIF2 occurs at Ser52, rather than Ser51, as observed in *S. cerevisiae* and mammalian eIF2α [33].

The three eIF2α kinases in *S. pombe* are regulated in response to different cellular stresses. Activation of Gcn2 occurs early after exposure to hydrogen peroxide (H_2_O_2_), methylmethanesulfonate (MMS), high concentrations of sodium chloride, 3-aminotriazole, or when cells are shifted from rich to minimal medium [33–38]. Hri2 is primary activated in response to heat shock, arsenite, cadmium, and glucose-depletion [33–35]. Compared to Gcn2 and Hri2, Hri1 is less well characterized but is activated at the onset of the stationary phase and under nitrogen-limiting conditions [34].

In the yeast *S. pombe*, a genetic approach to inducing heme-deficient conditions involves disrupting the *hem1*^*+*^ gene (*hem1*Δ), which encodes δ-aminolevulinic acid synthase (ALAS), the first enzyme in the biosynthetic pathway [39]. *S. pombe* cells lacking Hem1 activity can survive when supplemented with exogenous δ-aminolevulinate (ALA). The uptake of exogenous ALA allows heme biosynthesis to resume at the second enzymatic step and continue through the remaining biochemical reactions required for heme production. Alternatively, in the absence of ALA, *hem1*Δ cells can acquire heme by taking up exogenous hemin through their heme uptake machinery when cultured in a medium supplemented with exogenous hemin [40,41].

In this study, we used the *hem1*Δ-based approach to force cells undergoing a transition from ALA-sufficient to ALA-deficient conditions. RNA-seq data analysis identified 579 genes induced in response to ALA deprivation, including *hri1*^*+*^. Under this stress condition, Hri1 is the primary activated eIF2α kinase. Consistently, ALA starvation led to an increase in phosphorylated eIF2α levels, accompanied by a reduction in global protein synthesis. When strains lacking the different eIF2α kinases were grown under ALA-starved conditions, *hem1*Δ* hri1*Δ and *hem1*Δ* hri1*Δ* hri2*Δ* gcn2*Δ strains exhibited higher cell growth and global protein translation compared to *hem1*Δ *hri2*Δ and *hem1*Δ* gcn2*Δ mutant strains. In contrast, when strains subsequently undergo a transition from low ALA to hemin supplementation, *hem1*Δ* hri1*Δ and *hem1*Δ* hri1*Δ* hri2*Δ* gcn2*Δ strains displayed poor growth compared to *hem1*Δ* hri2*Δ and *hem1*Δ* gcn2*Δ strains. Taken together, these results revealed that in ALA-starved *hem1*Δ cells, Hri1 plays a crucial role in inhibiting global protein synthesis to mitigate stress caused by heme deficiency.

## Results

### During ALA deprivation, *hem1∆* cells experience a shortage of heme content.

**Fig 1:**
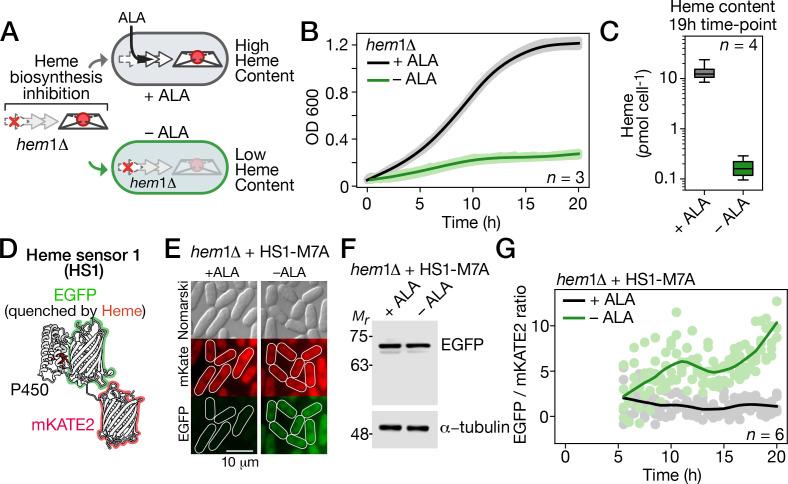
*ALA deprivation leads to a shortage of heme in hem1*∆ *cells.* A, Illustration of a *hem1∆*-based cellular system used to induce heme deficiency. In this system, endogenous heme production is arrested due to the deletion of Hem1 (*hem1∆*). Addition of exogenous δ-aminolevulinate (+ALA) restores heme biosynthesis at the second step of the pathway, maintaining high heme levels. In contrast, in the absence of exogenous ALA (-ALA), cellular heme levels are significantly depleted. B, *hem1∆* cells were precultured in YES medium containing ALA (25 µM). After washes, the cells were either incubated without ALA supplementation (-ALA) or supplemented with ALA (100 µM). Cell growth was monitored every 10 min for 19 h. The growth curves for three replicates are shown, with solid lines representing the mean values of the replicates. C, Heme content in *hem1∆* cells was quantified after 19 h of incubation in either ALA-free medium or medium supplemented with ALA. Total cellular heme content was measured using a fluorometric-based assay and expressed as picomoles of heme per cell. D, Three-dimensional model of heme sensor 1 (HS1) based on protein data bank (PDB) identification numbers 3BXB and 3U8P. E, Representative fluorescence images of mKATE (red) and EGFP (green) from HS1-M7A expressed in *hem1∆* cells after 19 h of incubation in either ALA-free medium or medium supplemented with ALA. Differential interference contrast microscopy (Nomarski) was used to examine cell morphology. The scale bar represents 10 µm. F, Whole cell extracts were prepared from aliquots of cultures described in panel E, and analyzed using immunoblot assays with anti-GFP and anti-α-tubulin antibodies. G, *hem1∆* cells expressing the heme sensor HS1-M7A were cultured as described in panel B. Changes in the EGFP/mKATE2 fluorescence ratio (green/red) for HS1-M7A were monitored over the indicated times. Dynamic variations in the EGFP/mKATE2 fluorescence ratio were depicted using the averages from six independent experiments.

The *hem1*^*+*^ gene, which encodes δ-aminolevulinate synthase, is essential for *S. pombe* survival [40]. Its disruption (*hem1*∆) is lethal unless exogenous δ-aminolevulinate (ALA) is supplied [40]. Supplementation with ALA allows heme biosynthesis to bypass the first enzymatic step and proceed through the subsequent reactions required for heme production (Fig 1A) [40,41]. In the absence of ALA, *hem1*∆ cells can be maintained alive if supplemented with exogenous hemin. Under these conditions, *hem1*∆ cells rely exclusively on their heme uptake machinery for growth. This experimental design (*hem1*∆ + hemin) has been employed to investigate the mechanisms by which *S. pombe* acquires exogenous heme [40–43]. Despite significant studies on heme acquisition, how *hem1*∆ cells respond to the shutdown of heme biosynthesis during ALA deprivation remains poorly understood (Fig 1A).

To further investigate the response to ALA starvation, *hem1∆* cells were precultured in the presence of ALA (25 µM) until reaching the mid-logarithmic phase. The cells were then washed and diluted to an OD_600_ of ~0.1. Subsequently, they were transferred to YES medium either without ALA supplementation or with ALA supplementation (100 µM) for 19 h. In the ALA-free medium, *hem1∆* cells exhibited poor growth, reaching an OD_600_ of 0.27 during this period (Fig 1B). In contrast, in the presence of ALA, *hem1∆* cells displayed robust growth, reaching an OD_600_ of 1.21 (Fig 1B). For each culture grown over a 19-h period, total heme levels were measured using a fluorometric assay that quantifies the fluorescence of protoporphyrin IX after iron dissociation [44–46]. In ALA-starved *hem1∆* cells, heme levels were measured at 0.18 pmol per cell, compared to 14.2 pmol per cell in ALA-replete *hem1∆* cells (Fig 1C).

To further validate cytosolic labile heme depletion in ALA-starved cells, we adapted the heme biosensor HS1-M7A for use in *S. pombe*. This biosensor is a variant of heme sensor 1 (HS1), which consists of the heme binding domain of Cyt-b562 fused to eGFP and the mKATE2 protein, enabling ratiometric fluorescence measurements (Fig 1D) [47,48]. Heme binding to Cyt-b_562_ quenches the GFP fluorescence without affecting mKATE2 fluorescence. Thus, the ratio of heme-sensitive eGFP fluorescence to heme-insensitive mKATE2 fluorescence provides a readout of cellular labile heme levels. When *hem1∆* cells expressing HS1-M7A were grown in the presence of exogenous ALA for 19 h, qualitative microscopy analysis revealed red fluorescence from mKATE2, while eGFP fluorescence was quenched, as no green signal was detected (Fig 1E). This ALA-replete condition suggests that endogenous heme synthesis generated sufficient cytosolic labile heme to quench eGFP fluorescence. In contrast, incubation of HS1-M7A-expressing *hem1∆* cells in ALA-free medium for 19 h resulted in the clear appearance of eGFP fluorescence, with no significant change in mKATE2 fluorescence (Fig 1E). This increase in eGFP fluorescence indicates a lack of labile heme, preventing eGFP fluorescence quenching. Notably, under both ALA-replete and ALA-depleted conditions, the steady-state levels of the heme sensor protein HS1-M7A remained comparable, exhibiting similar expression levels (Fig 1F).

Fluorometric assays were performed to monitor changes in the eGFP/mKATE2 fluorescence ratio as *hem1∆* cells were undergoing a transition from ALA-replete to ALA-starved conditions over 19 h. After washing and incubating HS1-M7A-expressing *hem1∆* cells in ALA-free medium for 5 h, eGFP/mKATE2 fluorescence ratio measurements were taken over the following 14 h. Results showed that under ALA-starved conditions, the eGFP/mKATE2 fluorescence ratio increased over time, reflecting reduced heme binding to the sensor and diminished labile heme levels. After 10 h, 15 h, and 19 h of incubation, the eGFP/mKATE2 fluorescence ratios increased by 62.7%, 70.8%, and 82.1%, respectively, compared to that of ALA-replete cells (Fig 1G). The highest eGFP/mKATE2 fluorescence ratio was measured at 19 h, the latest time-point examined, as prolonged ALA deprivation led to signs of cell sickness. In contrast, under ALA-replete conditions, the eGFP/mKATE2 fluorescence ratio remained consistently low over the time period of these experiments, indicating a stable pool of labile heme sufficient to quench eGFP fluorescence. Taken together, these findings reveal that *hem1∆* cells deplete their labile heme pool when undergoing a switch from ALA-replete to ALA-starved conditions.

### Transcriptome analysis of ALA-starved *hem1*∆ cells reveals up-regulation of stress-related genes, including the heme-regulated *hri1*^*+*^

**Fig 2:**
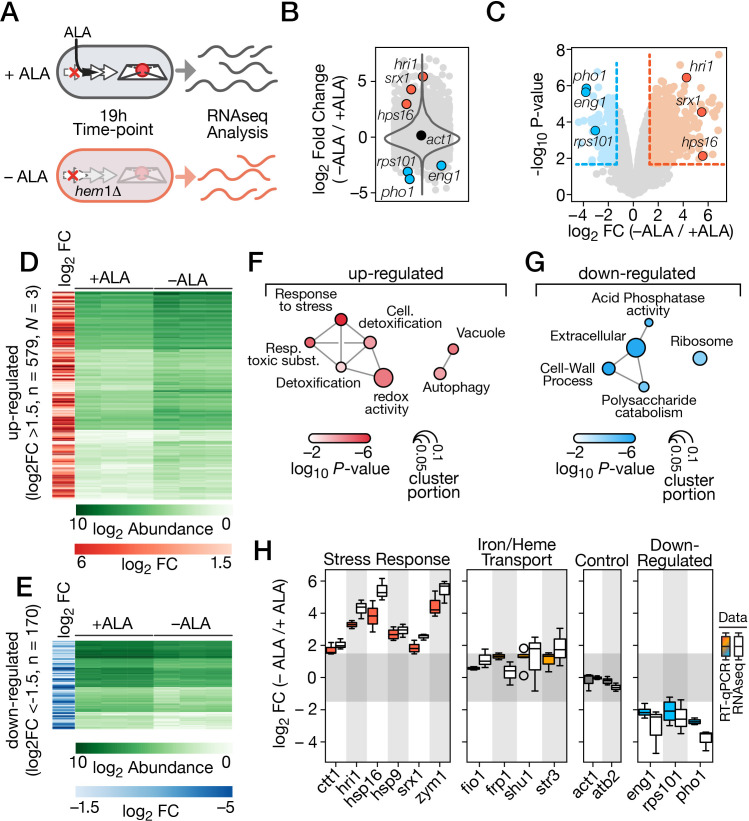
*Transcriptomic analysis of differentially expressed genes in ALA-starved versus ALA-replete hem1*Δ *cells.* A, Schematic representation of the experimental design for RNA-seq analysis. *hem1∆* cells precultured in the presence of ALA (25 µM) were washed and transferred to YES medium either supplemented with 100 µM ALA (+ALA) or left without ALA supplementation (-ALA) for 19 h. RNA was then extracted from each growth condition and used to generate RNA-seq librairies. B, Transcriptome-wide picture of the response to ALA starvation. Differentially expressed genes were analyzed in ALA-starved *hem1*Δ cells (-ALA) compared to ALA-replete *hem1*Δ cells (+ALA). A total of 5,780 transcripts were investigated by RNA-seq. While all differentially expressed genes are shown, only 7 are labelled on the graph. Examples of genes induced under low-ALA conditions are highlighted in red, whereas examples of genes repressed (blue) or unregulated (black) under the same conditions are also depicted. The y-axis represents the log_2_ fold change for each differentially expressed gene. C, Volcano plot showing significant differentially expressed genes in ALA-starved *hem1*Δ cells (-ALA; red) versus ALA-replete *hem1*Δ cells (+ALA; blue). The x-axis represents the log_2_ fold change, while the y-axis represents -log_10_ (P value) for each significantly differentially expressed gene. D, The heatmap in green exhibits 579 genes with higher expression levels in ALA-starved *hem1*Δ cells compared to ALA-replete *hem1*Δ cells. Darker green shades represent higher transcript abundance, whereas lighter green shades indicate lower transcript levels. The heatmap in red displays the average log_2_ fold change values in expression of the 579 genes induced in ALA-depleted cells (averaging >1.5 fold). E, The green heatmap displays 170 genes with lower expression levels in ALA-starved *hem1*Δ cells compared to ALA-replete *hem1*Δ cells. The blue heatmap shows the average log_2_ fold change values in expression of these 170 genes (averaging <1.5 fold). F - G, Gene ontology analysis of the genes up-regulated (red) or down-regulated (blue) under ALA deprivation conditions. Up-regulated genes encode proteins that are associated with different biological processes, including cellular stress response, protection against oxidative stress, and xenobiotic metabolism. In contrast, down-regulated genes are linked with pathways such as nutrient catabolism, ribosome biogenesis, and cell wall synthesis. H, Transcript log_2_ fold changes were calculated as the ratio of the expression levels of the indicated gene in ALA-starved *hem1*Δ cells compared to ALA-replete *hem1*Δ cells. Colored boxes (orange, gray, red, and blue) represent the quantification from three independent RT-qPCR assays, whereas empty boxes correspond to data analyzed from three separate RNA-seq experiments. Error bars indicate the standard deviation (± SD).

To further investigate the cellular responses upon inactivation of the heme biosynthetic pathway, we performed RNA-Seq transcriptomic analysis of *hem1*Δ cells using the following experimental design. Cells were precultured in the presence of 25 µM ALA, then washed, diluted and seeded to an OD_600_ of 0.1. One half of the cultures was incubated without ALA, whereas the other half was treated with ALA (100 µM) for 19 h. Total RNA was then isolated from each experimental condition and its respective replicates and used to construct RNA-Seq libraries (Fig 2A). The resulting sequencing reads were trimmed for adapters, low-quality 3’ ends, and a minimum length of 20 nucleotides. The read data were aligned to the *S. pombe* reference genome (*ASM294v2.25*) for gene annotations over a total of 5780 different genes. The log_2_-transformed fold-change (log_2_FC) in transcript abundance in ALA-starved compared to ALA-replete condition were considered for further analysis.

After verifying that data from replicates correlated with each other (S1 Appendix), we obtained a transcriptome-wide overview of *S. pombe hem1*Δ cells without ALA supplementation compared to those supplemented with ALA (Fig 2B). Positive log_2_FC values indicate genes with higher expression levels under ALA-starved conditions, whereas negative log_2_FC values correspond to genes with lower expression levels under these conditions (Fig 2B). Although the highest and lowest log_2_FC values ranged from 8.0 to -4.5, the overall distribution of log_2_FC values remained tightly centered around 0 (median = 0.006), indicating that the expression levels of many transcripts remained unchanged (Fig 2B). When log_2_FC values between the two conditions (-ALA/ + ALA) were plotted on the x-axis and the -log_10_ p-values on the y-axis, the resulting Volcano plot highlighted genes with decreased expression (log_2_FC < 1.5, left, blue) and genes with increased expression (log_2_FC > 1.5, right, red) (Fig 2C).

A total of 579 genes exhibited high expression levels in ALA-starved *hem1*Δ cells (Fig 2D). Gene ontology (GO) enrichment analysis revealed that some of these up-regulated genes encode stress-related proteins, including the heat shock proteins Hsp16, Hsp3102, and Hsp9, with log_2_FC expression levels of 5.42, 4.77, and 2.93, respectively (Fig 2D). Additionally, genes involved in oxidative stress defense, such as *srx1*^*+*^ and *ctt1*^*+*^ (log_2_FC of 2.54 and 2.06, respectively) were induced. Other up-regulated genes encode proteins associated with cellular detoxification processes, including Zym1, the formamidase-like SPAC869.04, and the hydroxyacid dehydrogenase-like SPAC186.07c (log_2_FC of 5.43, 3.97, and 3.95, respectively). Of particular interest, the *hri1*^*+*^ gene, which encodes heme-regulated inhibitor kinase 1, a member of the eIF2α kinase family, was highly expressed under ALA deprivation conditions (log_2_FC of 4.28) (Fig 2B-D).

In contrast, 170 genes exhibited decreased expression levels in ALA-starved *hem1*Δ cells (Fig 2E). Notably, genes encoding 5.8S ribosomal RNAs (*SPRRNA.50*, *SPRRNA.51*, and *SPRRNA.52*) and the 40S ribosomal protein S4 (*SPRRNA.04*) displayed log_2_FC repression levels of -3.10, -3.35, -2.97, and -2.57, respectively (Fig 2E). Other down-regulated genes included *agn1*^*+*^ and *eng1*^*+*^, which are involved in cell wall organization (log_2_FC of -2.28 and -3.10, respectively). The reduced transcription of these genes appears to correlate with decreased protein translation and impaired growth observed in *hem1*Δ cells under ALA deprivation conditions.

Following GO analysis (Fig 2F-G), we further examined whether the log_2_FC in transcript levels observed in RNA-Seq experiments aligned with transcript levels, as analyzed by RT-qPCR using independent biological replicates (Fig 2H). Under ALA-starved conditions, the expression of *hri1*^*+*^, *hsp16*^*+*^, *hsp9*^*+*^, *zym1*^*+*^, *srx1*^*+*^, and *ctt1*^*+*^ increased by 3.29-, 3.81-, 2.70-, 4.71-, 1.85-, and 1,77-log_2_FC, respectively, compared to levels measured under ALA-replete conditions in RT-qPCR assays (Fig 2H). These results were consistent with RNA-Seq data, where *hri1*^*+*^ (4.31-fold), *hsp16*^*+*^ (5.42-fold), *hsp9*^*+*^ (2.93-fold), *zym1*^*+*^ (5.43-fold), *srx1*^*+*^ (2.54-fold), and *ctt1*^*+*^ (2.06-fold) mRNA levels were induced (Fig 2H). For genes downregulated under ALA-starved conditions, *eng1*^*+*^, *rps101*^*+*^, and *pho1*^*+*^ exhibited a reduction of 2.11-, 2.10-, and 2.74-log_2_FC, respectively, in RT-qPCR assays compared to ALA-replete conditions (Fig 2H). Similarly, RNA-Seq analysis showed that *eng1*^*+*^, *rps101*^*+*^, and *pho1*^*+*^ transcript levels were repressed by 3.10-, 2.49-, and 3.82-log_2_FC, respectively (Fig 2H). Given that *hem1*∆ cells under ALA deprivation experience heme deficiency, we examined whether genes involved in inorganic iron (*fio1*^*+*^, *frp1*^*+*^) and heme (*shu1*^*+*^, *str3*^*+*^) transport were transcriptionally affected. Results indicated that *fio1*^*+*^, *frp1*^*+*^, *shu1*^*+*^, and *str3*^*+*^ expression was slightly induced under ALA-starved conditions compared to ALA-replete conditions (Fig 2H). However, their up-regulation was modest relative to *hri1*^*+*^, *hsp16*^*+*^, *hsp9*^*+*^, and *zym1*^*+*^, with log_2_FC increases of 0.60, 1.32, 1.22, and 1.64 for *fio1*^*+*^, *frp1*^*+*^, *shu1*^*+*^, and *str3*^*+*^, respectively, in RT-qPCR assays (Fig 2H). As controls, we monitored *act1*^*+*^ and *atb2*^*+*^ mRNA levels, which exhibited log_2_FC values of -0.18 and -0.02 for *act1*^*+*^ in RT-qPCR and RNA-Seq, respectively, while *atb2*^*+*^ showed log_2_FC values of -0.21 and -0.61 using the same methods (Fig 2H). Overall, log_2_FC values obtained from RNA-Seq experiments correlated well with those measured by RT-qPCR assays. Collectively, these findings identified 579 genes with increased expression in ALA-starved *hem1*Δ cells, while 170 genes exhibited reduced transcript abundance under the same conditions.

### ALA deficiency induces steady-state *hri1*^*+*^ transcript levels and activates the integrated stress response

**Fig 3:**
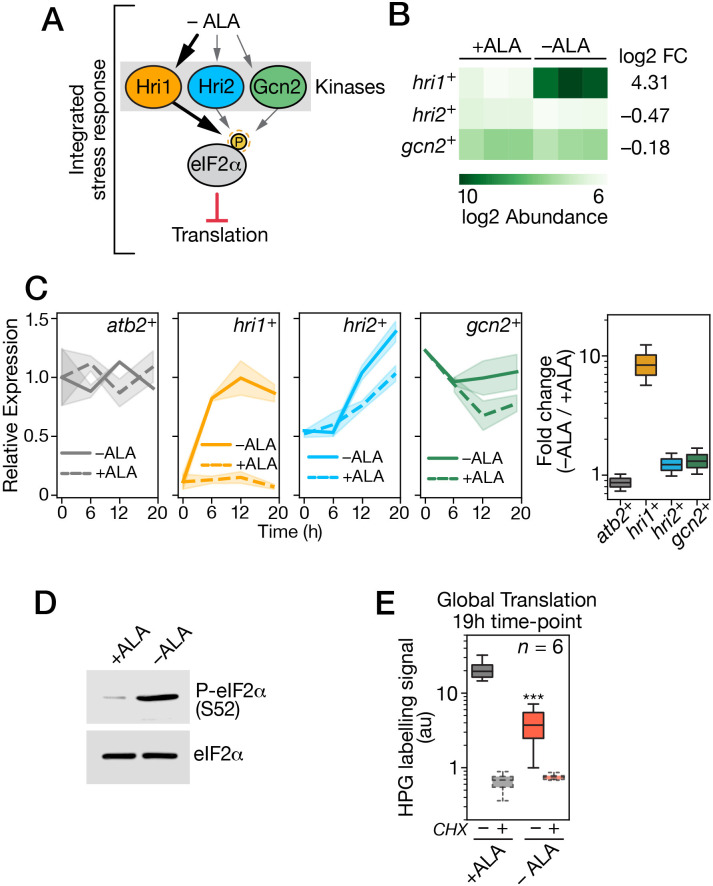
*Expression profiles of the hri1*^+^*, hri2*^+^*, and gcn2*^+^ *genes in ALA-starved hem1*Δ *cells.* A, Schematic representation of the signaling pathway involved in translational inhibition via phosphorylation of eIF2α by the Hri1, Hri2, and Gcn2 kinases in response to ALA deficiency. B, The green heatmap illustrates the transcriptional response from the RNA-seq data presented in Fig 2 for *hri1*^*+*^, *hri2*^*+*^, and *gcn2*^*+*^ mRNAs. Darker green shades indicate higher transcript abundance (log_2_). Log_2_ fold change values for the transcripts are shown on the right. C, *hem1∆* cells precultured in the presence of ALA (25 µM) were washed and then treated with 100 µM ALA (+ALA) or left without ALA supplementation (-ALA) for 0, 6, 12, and 19 h. Total RNA was isolated from culture aliquots collected at the indicated time points. Following RNA extraction, mRNA levels of *hri1*^*+*^, *hri2*^*+*^, *gcn2*^*+*^, and *atb2*^*+*^ were assessed by RT-qPCR assays. Transcript fold changes (*far-right bridge chart*) were calculated as the ratio of the expression levels of the indicated gene in ALA-starved cells compared to ALA-replete cells, and shown with gene color codes as follows: gray, *atb2*^*+*^; orange, *hri1*^*+*^; blue, *hri2*^*+*^; and green, *gcn2*^*+*^. D, Phosphorylation of eIF2α was detected in ALA-starved *hem1*Δ cells. At the 19-h time point, aliquots of the cultures used in *panel C* were taken to prepare whole cell extracts that were subjected to immunoblot assays. These assays utilized a polyclonal anti-eIF2α antibody and a monoclonal anti-phospho-eIF2α antibody. E, Assessment of global cellular translation in *hem1*Δ cells either treated with 100 µM ALA (+ALA, gray) or left without ALA supplementation (-ALA, red) for 19 h. In the final 30 min of treatment, HPG (10 µM) was added to cultures and then cells were fixed and permeabilized prior AlexaFluor647 azide fluorophore labelling of HPG-containing polypeptide chains. When indicated, cycloheximide (CHX, 0.1 mg/ml) was added to cultures 30 min prior HPG supplementation to inhibit translation. Bridge chart represents the quantification using the averages of values from six independent translation assays using Click-iT reactions. Error bars indicate the standard deviation (± SD).

We found that the *hri1*^*+*^ gene, which encodes an eIF2α kinase, is among the most highly induced genes in response to ALA deficiency in *hem1*Δ cells (Fig 2B-C). This gene particularly caught our attention as it was the only one among the three *S. pombe* genes encoding an eIF2α kinase to be significantly induced under ALA-starved conditions, with a log_2_FC value of 4.31 (Fig 3A-B). In contrast, after the same 19-h period of ALA starvation, *hri2*^*+*^ and *gcn2*^*+*^ transcript levels showed log_2_FC values of -0.47 and -0.18, respectively (Fig 3B).

We next assessed the temporal expression profiles of *hri1*^*+*^, *hri2*^*+*^, *gcn2*^*+*^, and *atb2*^*+*^ transcripts during ALA deprivation using RT-qPCR assays. *hri1*^*+*^ transcript levels were detected at 6, 12, and 19 h after *hem1*Δ cells were transferred to ALA-deprived conditions. The induction of *hri1*^*+*^ in response to ALA starvation was 5.3-fold at 6 h, 11.4-fold at 12 h, and 8.8-fold at 19 h compared to the corresponding time points under ALA-replete conditions (Fig 3C). For the *hri2*^*+*^ and *gcn2*^*+*^ transcripts, expression levels remained nearly unchanged under both experimental conditions over the 19-h period (Fig 3C). The *hri2*^*+*^ mRNA levels slightly varied by 0.8-, 1.3-, and 1.5-fold, whereas *gcn2*^*+*^ mRNA levels lightly fluctuated by 1.1-, 2.1-, and 1.7-fold under ALA-limiting conditions compared to ALA-replete conditions (Fig 3C). The *atb2*^*+*^ transcript, which encodes α-tubulin, served as an internal control in these RT-qPCR assays (Fig 3C).

Although all three *S. pombe* eIF2α kinases were expressed under conditions of ALA starvation, only the *hri1*^*+*^ gene, which encodes Hri1, was markedly induced in response to ALA deprivation, suggesting its specific activation by this stimulus (Fig 3B-C). We next examined whether ALA deprivation triggered phosphorylation of the α subunit of eIF2 to inhibit protein synthesis through the common adaptive pathway known as the integrated stress response (ISR) [19–23]. In *S. pombe*, eIF2α is phosphorylated at the highly conserved Ser52, which corresponds to position 51 in *S. cerevisiae* and mammalian eIF2α orthologs [33,34]. *hem1∆* cells were precultured in medium containing ALA (25 µM). After washing and dilution, the cells were incubated in YES medium either with ALA supplementation (100 µM) or without ALA supplementation for 19 h. Whole cell extracts were then prepared and analyzed by immunoblotting. Levels of eIF2α phosphorylation in *hem1∆* cells were assessed using an antibody specific to phosphorylated eIF2α. The results showed that phosphorylated eIF2α levels were markedly lower under ALA-replete conditions compared to ALA-limiting conditions, where the phosphorylated form was abundant (Fig 3D). In contrast, the levels of total eIF2α remained similar under both ALA-replete and ALA-depleted conditions (Fig 3D). These results indicated that eIF2α was phosphorylated at Ser52 during ALA deprivation in *hem1∆* cells.

During the ISR, studies have shown that phosphorylation of eIF2α on Ser51 (or Ser52 in *S. pombe*) inhibits the guanine nucleotide exchange factor eIF2B, leading to a general reduction in protein synthesis [20,22,23,38]. To assess global cellular translation, *hem1∆* cells were incubated in ALA-free medium either without ALA supplementation or with ALA supplementation for 19 h. In the final 30 min of incubation, alkyne-labelled L-homoproparglyglycine (HPG), a methionine analogue, was added to monitor its incorporation into newly synthesized proteins. After fixation and permeabilization, cultures were treated with an AlexaFluor azide fluorophore, which covalently attaches to HPG-labelled proteins through Click azide-alkyne reactions [49,50]. Results showed that ALA-starved hem1∆ cells exhibited a 5.4-fold reduction in HPG incorporation compared to cells incubated under ALA-replete conditions (Fig 3E). As a control, cycloheximide (CHX), a translation inhibitor, was added to parallel cultures 30 min prior HPG treatment. Consistently, CHX-treated cultures displayed minimal HPG incorporation, confirming the inhibition of translation. Specifically, CHX treatment resulted in 32.1-fold and 5.3-fold reductions in HPG incorporation compared to untreated cells under ALA-replete and ALA-deficient conditions, respectively (Fig 3E). Taken together, these results showed that *hri1*^*+*^ emerges as the primary eIF2α kinase gene exhibiting a marked increase in steady-state transcript levels in response to ALA deprivation. Under these conditions, phosphorylated eIF2α levels increase, leading to a global decrease in protein translation.

### Effect of Hri1 deletion or the inactivation of all three eIF2α kinases on the growth of *hem1∆* cells in ALA-deficient medium, with or without hemin supplementation

**Fig 4:**
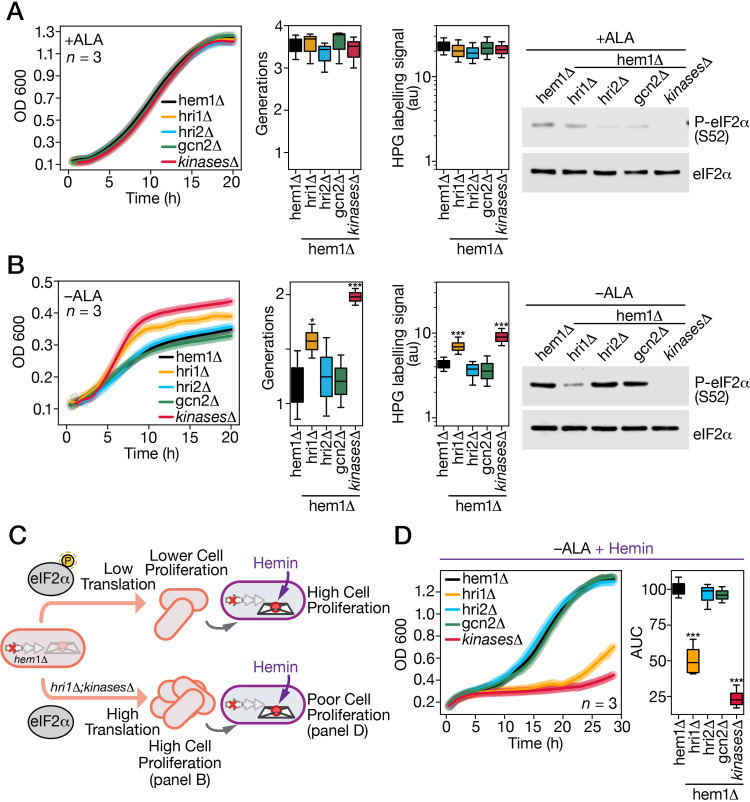
*Disruption of hri1*+ *leads to reduced phosphorylation of eIF2*α *and impairs the recovery process from ALA starvation upon hemin supplementation.* A, The indicated strains were grown in YES medium supplemented with ALA (25 µM). The growth of the strains was monitored at the indicated time points (*far-left graph*). Strain color codes are as follows: black, *hem1*Δ; orange, *hem1*Δ* hri1*Δ; blue, *hem1*Δ* hri2*Δ; green, *hem1*Δ* gcn2*Δ; red, *hem1*Δ* hri1*Δ* hri2*Δ* gcn2*Δ (*kinases*Δ). For the next graph, its y-axis represents the number of generations for the indicated strains, calculated based on the total number of cell population doublings over a period of 19 h. The middle-right graph represents the assessment of global cellular translation in the indicated strains grown in the presence of ALA for 19 h. In the final 30 min of proliferation, HPG (10 µM) was added to cultures prior AlexaFluor647 azide fluorophore labelling of HPG-containing polypeptide chains. Bridge chart represents the quantification using the averages of values from three independent translation assays using Click-iT reactions. Error bars indicate the standard deviation (± SD). At the 19-h time point, aliquots of the cultures were taken to prepare whole cell extracts that were subjected to immunoblot assays. These assays utilized a polyclonal anti-eIF2α antibody and a monoclonal anti-phospho-eIF2α antibody. B, the specified strains were precultured in YES medium containing ALA (25 µM). After washes, the strains were incubated without ALA supplementation (-ALA). Cell growth was monitored every 10 min for 19 h (*far-left graph*). Solid lines represent the mean values of three replicates. The second graph exhibits measurement of cell population doublings over a period of 19 h. Results are shown as averages ± SD of three independent experiments that were performed in biological triplicate. The asterisks correspond to p < 0.1 (*), and p < 0.001 (***) (one-way ANOVA with Dunnett’s multiple comparisons test against ALA-starved hem1Δ cells). The third graph represents the assessment of global cellular translation in the indicated strains incubated in YES medium without ALA supplementation for 19 h. In the final 30 min of incubation, HPG (10 µM) was added to cultures as described in *panel A*, and its incorporation into proteins, detected using Click-iT chemistry, served as a measure of global cellular translation. At the 19-h time point, aliquots of the indicated strains grown in ALA-deprived media were used to prepare whole cell extracts that were subjected to immunoblot assays. These assays utilized a polyclonal anti-eIF2α antibody and a monoclonal anti-phospho-eIF2α antibody. C, Schematic representation illustrating the hemin-dependent growth defect of the *hem1*Δ *hri1*Δ and *hem1*Δ* hri1*Δ* hri2*Δ* gcn2*Δ (*hem1*Δ* kinases *Δ) mutant strains following their culture in an ALA-deficient medium (*panel* D), where a reduction or absence of eIF2α phosphorylation was observed, respectively. D, The indicated strains were incubated without ALA supplementation (-ALA) for 19 h as described in *panel* B. At this stage, strains were diluted to an OD_600_ of 0.1 (zero time point) and then incubated in ALA-free medium supplemented with hemin (1 µM) where cell growth was monitored by measuring optical cell density (*left graph*). The area under the curve (AUC) for each strain’s growth curve was calculated and plotted in the bridge chart (*right graph*). Results represent averages ± SD from three independent experiments performed in biological triplicate. The asterisks correspond to p < 0.001 (***) (one-way ANOVA with Dunnett’s multiple comparisons test against ALA-starved hem1Δ cells).

Based on the transcriptional activation of *hri1*^*+*^ gene expression in response to ALA deprivation, we next investigated whether the deletion of Hri1 affected the growth of *hem1∆* cells in ALA-deficient medium. First, *hem1∆ hri1∆*, *hem1∆ hri2∆*, *hem1∆ gcn2∆*, *hem1∆ hri1∆ hri2∆ gcn2∆* (*kinases ∆*) strains were incubated in the presence of ALA to verify that their growth was comparable to the *hem1∆* parental strain (Fig 4A). After 19 h of proliferation, all strains exhibited a similar number of generations, indicating equivalent doubling rates (Fig 4A). To assess global translation levels, cultures were grown in parallel experiments and incubated with HPG during the last 30 min of proliferation. HPG-treated cells were stained with an AlexaFluor azide fluorophore, enabling the detection of HPG incorporation into newly synthesized proteins via Click Chemistry. The results showed that global translation levels, as measured by HPG incorporation, were comparable across all five strains under ALA-replete conditions (Fig 4A). We analyzed eIF2α phosphorylation levels in the five strains cultured under non-stress, ALA-replete conditions. Western blot analysis revealed low phosphorylation levels of eIF2α at Ser52 in *hem1∆* and *hem1∆ hri1∆* strains, whereas phosphorylation was even lower in *hem1∆ hri2∆* and *hem1∆ gcn2∆* strains. As a control, no phosphorylation was detected in the strain lacking all three kinases (Fig 4A).

Following preculture in the presence of ALA (25 µM), *hem1∆*, *hem1∆ hri1∆*, *hem1∆ hri2∆*, *hem1∆ gcn2∆*, and *hem1∆ hri1∆ hri2∆ gcn2∆* (*kinases ∆*) strains were washed, diluted, and then seeded in ALA-free medium to monitor their cell proliferation. Surprisingly, the *hem1∆ hri1∆ hri2∆ gcn2∆* quadruple mutant exhibited 20–28% and 19–26% more growth than the *hem1∆*, *hem1∆ hri2∆*, and *hem1∆ gcn2∆* strains after 12 and 19 h, respectively (Fig 4B). Similarly, the *hem1∆ hri1∆* mutant exhibited 10-17.5% more growth than the *hem1∆*, *hem1∆ hri2∆*, and *hem1∆ gcn2∆* strains after 12 h; however, its growth was 10% lower than that of the quadruple mutant (*kinases ∆*) (Fig 4B). After 19 h of proliferation in ALA-deficient medium, growth assays showed that the quadruple mutant strain produced 1.98 cell generations, whereas the *hem1∆ hri1∆* mutant produced 1.58 generations (Fig 4B). In contrast, the *hem1∆*, *hem1∆ hri2∆*, and *hem1∆ gcn2∆* mutant strains exhibited lower generation numbers, with 1.17, 1.24, and 1.20 generations, respectively (Fig 4B). The aforementioned strains, cultured in ALA-free medium, were treated with HPG during the final 30 min of growth. AlexaFluor azide fluorophore was then added to detect HPG incorporation into de novo synthesis of polypeptides. The results showed that the quadruple mutant (*kinases ∆*) exhibited the highest HPG incorporation signal, with 9.24 units of fluorescence detected by Click-iT assays (Fig 4B). Consistent with the cell proliferation assays, the *hem1∆ hri1∆* strain displayed the second highest HPG incorporation signal in newly synthesized proteins, with 7.10 units (Fig 4B). In contrast, the *hem1∆*, *hem1∆ hri2∆*, and *hem1∆ gcn2∆* mutant strains exhibited lower HPG incorporation signals, with values of 4.36, 3.65, and 3.86 units, respectively (Fig 4B). Given that global cellular translation was reduced in *hem1∆*, *hem1∆ hri2∆*, and *hem1∆ gcn2∆* strains, we examined the levels of eIF2α phosphorylation in these mutants. Western blot analysis showed high phosphorylation levels of eIF2α at Ser52 in *hem1∆*, *hem1∆ hri2∆*, and *hem1∆ gcn2∆* strains (Fig 4B). In contrast, low phosphorylation levels were detected in *hem1∆ hri1∆* cells grown under ALA-limiting conditions (Fig 4B). As a control, eIF2α phosphorylation was impaired in the strain lacking all three kinases (Fig 4B).

After 19 h in ALA-free medium, *hem1∆*, *hem1∆ hri1∆*, *hem1∆ hri2∆*, *hem1∆ gcn2∆*, and *hem1∆ hri1∆ hri2∆ gcn2∆* cells were diluted to an OD_600_ of 0.1 and then incubated in ALA-deficient medium supplemented with exogenous hemin (1 µM) for 28.5 h (Fig 4C). The results showed that *hem1∆*, *hem1∆ hri2∆*, and *hem1∆ gcn2∆* strains exhibited robust cell growth, reaching OD_600_ values of 1.05, 1.09, and 1.03, respectively, after 20 h (Fig 4D). In contrast, *hem1∆ hri1∆* and *hem1∆ hri1∆ hri2∆ gcn2∆* strains displayed poor growth, with OD_600_ values of 0.35 and 0.33, respectively, over the same period (Fig 4D). In the case of *hem1∆ hri1∆* cells, they exhibited a noticeable increase in growth (OD_600_ from 0.41 to 0.69) during the last 6.5 h, compared to the strain lacking all three kinases (Fig 4D).

The area under the curve (AUC) for each growth curve was determined to facilitate comparisons between the mutant strains (Fig 4D). The *hem1∆* strain exhibited the highest AUC value, which was arbitrarily set to 100. Given their similar growth phenotypes, the *hem1∆ hri2∆* and *hem1∆ gcn2∆* strains displayed AUC values of 96.15 and 95.16, respectively (Fig 4D). Consistently, the *hem1∆ hri1∆* and *hem1∆ hri1∆ hri2∆ gcn2∆* strains exhibited the lowest AUC values, at 50.81 and 23.83, respectively, compared to the *hem1∆* control strain (Fig 4D). Collectively, these results revealed that a global reduction in protein synthesis in response to ALA deficiency is critical for *S. pombe hem1∆* cells to successfully resume growth upon the addition of exogenous hemin to the cultures. Furthermore, Hri1 is the primary kinase responsible for eIF2α phosphorylation in response to ALA deficiency in *S. pombe*.

### Phosphorylation of eIF2α at Ser52 by Hri1 is sufficient to withstand ALA shortage stress

**Fig 5:**
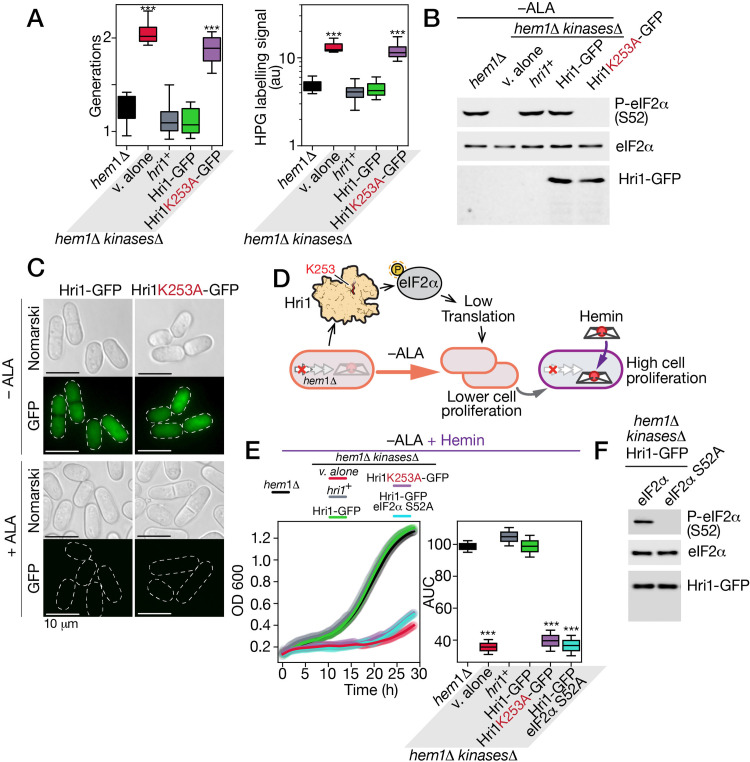
*Effect of Lys*^*253*^*Ala mutation on the ability of Hri1 to phosphorylate eIF2*α. A, The indicated strains were incubated in ALA-free medium for 19 h, and cell population doublings were measured during this period (*left graph*). Strain color codes are as follows: black, *hem1*Δ; red, *hem1*Δ* hri1*Δ* hri2*Δ* gcn2*Δ (*kinases*Δ); gray, *hem1*Δ* kinases*Δ expressing *hri1*^*+*^; green, *hem1*Δ* kinases*Δ expressing *hri1*^*+*^*-GFP*; violet, *hem1*Δ* kinases*Δ expressing *hri1K253A-GFP*. In the final 30 min of incubation in ALA-free medium, strains were treated with HPG (10 µM), then fixed and permeabilized for labelling of HPG-containing polypeptides using AlexaFluor647 azide fluorophore. The bridge chart (*right*) shows the quantification of translation using the averages of values from three independent assays performed in biological triplicate using Click-iT reactions. Error bars indicate the standard deviation (± SD). B, Whole-cell extracts were prepared from aliquots of cultures described in panel A. Samples were analyzed by immunoblotting using anti-GFP, anti-eIF2α and anti-phospho-eIF2α antibodies. C, *hem1*Δ* hri1*Δ* hri2*Δ* gcn2*Δ cells expressing *hri1*^*+*^*-GFP* or *hri1K253A-GFP* under the control of their own promoters were incubated in YES medium supplemented with ALA (25 µM) or lacking ALA for 19 h. Cells were analyzed by fluorescence microscopy to detect GFP-dependent fluorescence signals (lower panels of each pair). Nomarski optics was used to examine cell morphology. D, A schematic representation illustrates Hri1-dependent phosphorylation of eIF2α when cells were incubated in ALA-deficient medium prior their exposure to exogenous hemin. E, After culturing in ALA-deficient medium, *hem1*Δ* hri1*Δ* hri2*Δ* gcn2*Δ cells expressing functional *hri1*^*+*^ (gray) or *hri1*^*+*^*-GFP* (green) alleles restored growth in the presence of exogenous hemin as the sole source of heme. In contrast, cells expressing the *hri1K253A* (red) or eIF2αS52A (blue) mutant allele exhibited poor grow under these conditions (*left graph*). The area under the curve (AUC) for each strain’s growth curve was determined and shown in the bridge chart (*right graph*). Results are shown as averages ± SD of three independent experiments performed in biological triplicate. F, Whole-cell extracts from the indicated strains were analyzed by immunoblot assays using anti-GFP, anti-eIF2α and anti-phospho-eIF2α antibodies.

To further investigate the role of Hri1 in promoting cellular recovery from ALA deprivation stress, we used the *hem1∆ hri1∆ hri2∆ gcn2∆* quadruple mutant strain and reintegrated either an untagged *hri1*^*+*^, a GFP-tagged *hri1*^*+*^, or a GFP-tagged *hri1K253A* mutant allele. As a control, an empty vector was also integrated into *hem1∆ hri1∆ hri2∆ gcn2∆* cells. All transformed cells were precultured in the presence of ALA and then transferred to an ALA-free medium, where their growth was monitored over 19 h. As expected, the quadruple mutant strain harboring an empty vector exhibited the highest number of generations, with 2.10 doublings (Fig 5A). Similarly, *hem1∆ hri1∆ hri2∆ gcn2∆* cells expressing Hri1K253A-GFP also displayed a high generation number, with 1.87 doublings (Fig 5A). In contrast, *hem1∆ hri1∆ hri2∆ gcn2∆* cells expressing either the untagged *hri1*^*+*^ or *hri1*^*+*^*-GFP* allele exhibited significantly lower generation numbers, with 1.16 and 1.11 doublings, respectively (Fig 5A). As a control, the parental *hem1∆* strain, which expresses endogenous eIF2α kinases, displayed a similarly low generation number of 1.23 doublings (Fig 5A).

Consistently, we found that *hem1∆ hri1∆ hri2∆ gcn2∆* cells expressing either an empty vector or the *hri1K253A* allele, which encodes a kinase-deficient mutant, exhibited the highest levels of HPG incorporation, with fluorescence signals of 13.55 and 12.35 units, respectively, as detected by Click-iT assays (Fig 5B). These results indicated a lack of global protein synthesis reduction in these strains under ALA deprivation. In contrast, when Click-iT assays were performed on *hem1∆* and *hem1∆ hri1∆ hri2∆ gcn2∆* cells expressing a functional *hri1*^*+*^ or *GFP-tagged hri1*^*+*^ allele under ALA starvation conditions, a significant decrease in global protein synthesis was observed, as reflected by lower HPG incorporation signals, with values of 4.90, 4.13, and 4.55 units, respectively (Fig 5A).

Next, we examined eIF2α phosphorylation levels in ALA-starved *hem1∆ hri1∆ hri2∆ gcn2∆* cells expressing either untagged *hri1*^*+*^ or *hri1*^*+*^*-GFP* under the control of their native promoter. Immunoblot analysis was performed using an antibody that specifically recognizes the phosphorylated form of eIF2α at Ser52 (in *S. pombe*). Phosphorylated eIF2α was detected in whole cell extracts from cells expressing Hri1 and Hri1-GFP (Fig 5B). In contrast, no phosphorylated eIF2α was observed in *hem1∆ hri1∆ hri2∆ gcn2∆* cells harboring either an empty vector or the *hri1K253A-GFP* allele, which encodes a kinase-deficient mutant (Fig 5B). As a control, Ser52 phosphorylation of eIF2α was detected in ALA-starved *hem1∆* cells expressing the endogenous *hri1*^*+*^ gene (Fig 5B). Parallel immunoblot analysis of cell extracts from culture aliquots showed the production of Hri1-GFP and Hri1K253A-GFP fusion proteins under conditions of ALA starvation (Fig 5B).

As shown in Fig 2, *hri1*^*+*^ mRNA levels were induced 19.4-fold (log_2_FC of 4.28) upon ALA starvation but repressed under ALA-replete conditions. Fluorescence microscopy revealed that the Hri1-GFP fluorescent signal mirrored *hri1*^*+*^ mRNA expression, being primarily detected under ALA deprivation conditions (Fig 5C). In contrast, the Hri1-GFP fluorescence signal was lost when *hem1∆ hri1∆ hri2∆ gcn2∆* cells harboring a *hri1*^*+*^*-GFP* allele were incubated under ALA-replete conditions (Fig 5C). Under ALA-limiting conditions, Hri1-GFP fluorescence signal was primarily observed throughout the cytoplasm (Fig 5C). Similarly, the kinase-deficient Hri1K253A-GFP mutant localized to the cytoplasm in ALA-starved *hem1∆ hri1∆ hri2∆ gcn2∆* cells but lost its fluorescence signal when cells were grown in the presence of ALA (Fig 5C).

After 19 h of ALA deprivation, *hem1∆* and *hem1∆ hri1∆ hri2∆ gcn2∆* cells expressing Hri1, Hri1-GFP, or the kinase-deficient mutant Hri1K253A-GFP were transferred and seeded at an OD_600_ of 0.1 in ALA-free medium supplemented with exogenous hemin (1 µM) for 28.5 h (Fig 5D-E). As controls, *hem1∆ hri1∆ hri2∆ gcn2∆* cells carrying an empty vector and *hem1∆ hri1∆ hri2∆ gcn2∆* cells expressing *hri1*^*+*^*-GFP* and *eIF2αS52A* (also called *tif211S52A*) alleles were cultured under the same conditions. Cell proliferation assays showed that *hem1∆ hri1∆ hri2∆ gcn2∆* cells expressing Hri1 and Hri1-GFP reached OD_600_ values of 1.27 and 1.29, respectively, after 28.5 h (Fig 5E). This level of growth was comparable to that of *hem1∆* cells expressing the endogenous *hri1*^*+*^ gene, which reached an OD_600_ of 1.26 after 28.5 h (Fig 5E). In contrast, *hem1∆ hri1∆ hri2∆ gcn2∆* cells expressing the kinase-deficient Hri1K253A-GFP mutant exhibited poor growth, with OD_600_ values of 0.28 and 0.51 after 20 and 28.5 h, respectively (Fig 5E). A similar growth defect was observed in *hem1∆ hri1∆ hri2∆ gcn2∆* cells carrying an empty vector, which had OD_600_ values of 0.24 and 0.39 after 20 and 28.5 h, respectively (Fig 5E). As an additional control, *hem1∆ hri1∆ hri2∆ gcn2∆* cells expressing Hri1-GFP along with a mutant form of eIF2α in which Ser52 was substituted with alanine also showed poor growth, with OD_600_ values of 0.25 and 0.48 at the same time points (Fig 5E).

To make easier comparisons between strains, the AUC for each growth curve was measured (Fig 5E). The highest AUC values were observed in *hem1∆* and *hem1∆ hri1∆ hri2∆ gcn2∆* cells expressing Hri1 and Hri1-GFP, with scores of 98.7, 104.8, and 99.0, respectively (Fig 5E). In contrast, *hem1∆ hri1∆ hri2∆ gcn2∆* cells lacking all three kinases or expressing the mutant Hri1K253A displayed significantly lower AUC values of 36.0 and 39.6, respectively, compared to the *hem1∆* control strain (Fig 5E). Similarly, *hem1∆ hri1∆ hri2∆ gcn2∆* cells expressing Hri1-GFP along with the eIF2αS52A mutant also showed a low AUC value of 36.5. In this latter strain, immunoblot analysis confirmed the absence of phosphorylated eIF2α when Ser52 was substituted with alanine (Fig 5F). Taken together, these results highlighted the critical role of Hri1 in participating in cellular recovery from ALA deprivation stress upon hemin supplementation.

### Hri1 exhibits high-affinity binding to hemin

**Fig 6:**
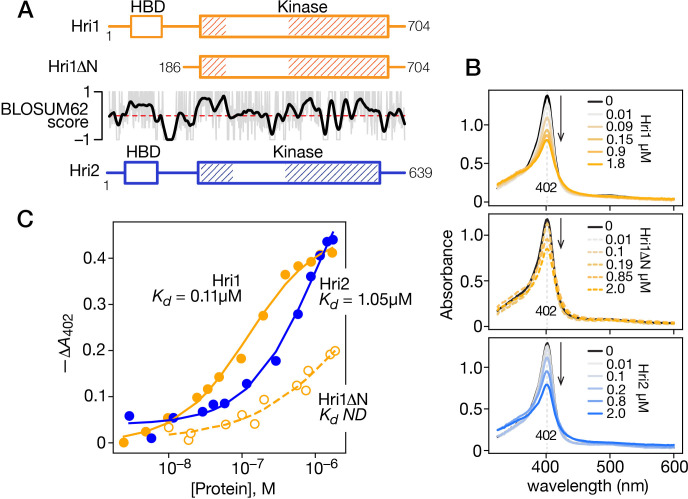
Hri1 and its paralog Hri2 interact with hemin. A, Schematic representation of Hri1, Hri2, and the Hri1ΔN mutant. A positive BLOSUM62 score indicates regions of the proteins with conserved amino acid residues, whereas a negative score displays non-conserved residues between Hri1 and Hri2 proteins. The predicted heme-binding domain (HBD) spans residues 44-119 in Hri1 and residues 37-112 in Hri2. The predicted protein kinase domain includes conserved subdomains, shown as hatched regions, covering residues 224-291 and 433-662 in Hri1, and residues 172-252 and 360-583 in Hri2. B, Wild-type Hri1, Hri2, and the Hri1ΔN mutant were expressed in *E. coli*, purified, and analyzed via differential spectral titration using 5 µM hemin. Titration assays were performed with the indicated increasing concentrations of Hri1, Hri2, or Hri1ΔN. Absorbance changes at the Soret peak (402 nm) were plotted against protein concentrations to generate binding curves. C, Wild-type Hri1 and Hri2 exhibited K_D_ values of 0.11 × 10^-6^ M and 1.05 × 10^-6^ M, respectively. In contrast, the Hri1ΔN mutant showed minimal interaction with hemin, precluding the determination of a *K*_*D*_ value.

Our transcriptomic data revealed that among the three genes encoding *S. pombe* eIF2α kinases, *hri1*^*+*^ is the most highly induced under ALA-deprived conditions. These results strongly suggested that Hri1 is the primary kinase activated in response to ALA deficiency. In contrast, when *hem1∆* cells were cultured under ALA-replete conditions, heme biosynthesis resumes at the second enzymatic step and proceeds further along the pathway until heme is produced. Under these conditions, not only was the *hri1*^*+*^ gene repressed, but the pre-existing Hri1 protein would also be inactivated through its binding to heme. To further investigate the heme-binding capacity of Hri1, wild-type Hri1, Hri2, and a mutated version of Hri1 were expressed in *E. coli* (Fig 6A). The mutant form, denoted Hri1ΔN, was generated by deleting the first 185 residues (Fig 6A). MBP-tagged wild-type Hri1, Hri2, and the Hri1ΔN mutant were purified to near homogeneity using two rounds of affinity chromatography based on MBP affinity for maltose. Before using these purified proteins for spectroscopic analysis, we confirmed that a typical reaction containing hemin, without the addition of protein, exhibited an absorption peak at 402 nm, corresponding to the Soret peak (Fig 6B). When increasing concentrations of wild-type Hri1 were added to a constant hemin concentration (5 µM), the protein triggered a reduction in hemin absorbance as a function of increasing its concentrations (Fig 6B). Similarly, the addition of wild-type Hri2 fostered a comparable reduction of the Soret peak, although at very low Hri2 concentrations, only a slight decrease in absorbance at 402 nm was observed (Fig 6B). In contrast to wild-type Hri1, addition of the Hri1ΔN mutant failed to produce a significant decrease in the Soret peak intensity (Fig 6B). Analysis of absorbance changes at 402 nm as a function of wild-type Hri1 and Hri2 concentrations showed that these proteins bound hemin with *K*_*D*_ values of 0.11 × 10^-6^ M and 1.05 × 10^-6^ M, respectively (Fig 6C). In contrast, the lack of significant interaction between Hri1ΔN and hemin prevented determination of its *K*_*D*_ value (Fig 6C). Taken together, these results revealed that Hri1 interacts with hemin with a *K*_*D*_ of one order of magnitude greater than that of Hri2, highlighting its exquisite heme sensitivity.

## Discussion

In this study, we investigated the transcriptional regulation of gene expression in response to ALA deprivation in *S. pombe* under conditions where its endogenous heme biosynthesis machinery was non-functional. Notably, we found that the *hri1*^*+*^ gene, which encodes one of the three eIF2α kinases, was strongly induced under this stress. The function of Hri1 remains largely unknown. ALA starvation prevents the synthesis of new heme, which, in its absence, cannot bind to and inhibit Hri1, leading to its activation. Once activated, Hri1 phosphorylates the translation initiation factor eIF2α, triggering the integrated stress response (ISR) and leading to a global reduction in protein translation [19,21,31,33,34]. Conversely, in parallel, the translation of specific transcription factors is enhanced, promoting the transcriptional activation of several stress response genes [51,52]. Consistent with this, our RNA-Seq analysis revealed that several stress-responsive genes were induced after 19 h of ALA deprivation (Fig 2D). For instance, the transcription levels of *ctt1*^*+*^, *srx1*^*+*^, and *yhb1*^*+*^, which encode catalase, sulfiredoxin, and nitric oxide dioxygenase, respectively, were upregulated. This suggests an increase in reactive oxygen species (ROS) production under ALA starvation conditions. This observation is reminiscent of a previous screen in which *S. pombe* mutant strains were identified based on their hypersensitivity to oxidative stress induced by hydroxyurea [53]. One such mutant, denoted *hem13–1*, was defective in heme biosynthesis and exhibited abnormally elevated ROS levels. This finding aligns with our observations, indicating that disruption of heme production promotes oxidative stress. An explanation for this phenomenon is that the majority of iron taken up by yeast cells is utilized by mitochondria, primarily for heme biosynthesis and the assembly of iron-sulfur clusters [54]. When heme biosynthesis is disrupted, an increased pool of labile iron may accumulate, potentially fueling Fenton redox reactions. This, in turn, could lead to the production of radical species, ultimately causing oxidative stress-induced damage to cellular components. Supporting this hypothesis, the oxidative stress sensitivity of the *hem13–1* mutant was largely suppressed by treatment with the iron chelator 2,2’-bipryidine [53].

Previous studies have demonstrated a role for the eIF2α kinases Gcn2 and Hri2, as well as the mitogen-activated protein kinase Sty1, in hydrogen peroxide-mediated oxidative stress response in fission yeast [33]. Although Gcn2 is the primary eIF2α kinase activated in the early stages of oxidative stress, Sty1 is also activated and exerts a negative effect on Gcn2 and Hri2 during prolonged oxidative stress exposure [33]. It is hypothesized that this negative regulation by Sty1 helps maintain a minimal level of eIF2α activity under stress conditions, allowing the translation of specific mRNAs essential for cell survival. However, it remains unknown whether Sty1 plays a role in modulating Hri1 in response to ALA deprivation, and this aspect requires further investigation.

Unlike oxidative stress induced by hydrogen peroxide, previous studies have shown that Sty1 promotes the rapid activation of Gcn2 in response to histidine starvation triggered by the drug 3-aminotriazole (3AT) [36,37]. In the absence of Sty1 (*sty1*Δ), phosphorylation of the eIF2α kinase Gcn2 is significantly delayed during the first 30 min of 3AT treatment, thereby impairing the proper activation of the Gcn2-dependent stress response pathway.

Although Gcn2 is a key eIF2α kinase involved in regulating the integrated stress response (ISR) during histidine starvation, Hri1 and Hri2 have been shown to partially compensate for the loss of Gcn2 in response to 3AT. In this context, a set of genes (referred to as cluster 5) is upregulated to help mitigate the effects of histidine starvation [37]. However, a comparison between this gene cluster, which includes genes predicted to function in protein folding, degradation or mitochondrial maintenance, and the 579 genes induced in ALA-starved *hem1*Δ cells (where *hri1*^*+*^ is highly expressed) revealed non-significant overlap, with only 5 genes in common. This observation suggests that the eIF2α kinase Hri2 may be more closely associated with the Gcn2-dependent response to histidine starvation.

In this study, we found that the reduction of general protein translation in response to ALA depletion is primarily mediated by Hri1. In *S. pombe*, a similar down-regulation of general translation is observed under amino acid starvation conditions [55]. In this case, the ISR is primarily transduced by the eIF2α kinase Gcn2. This reduction in general translation is accompanied by an increased translation of specific mRNAs, including the transcript encoding the transcription factor Fil1. Fil1, in turn, activates the expression of genes involved in amino acid biosynthesis to remediate for amino acid deficiency [55]. At this stage, it remains unclear whether Fil1 is involved in the Hri1-eIF2α signaling response to ALA deprivation. Its potential role requires further investigation.

Interestingly, in *S. cerevisiae* and mammals, the translation of the transcription factors Gcn4 and Atf4, respectively, is induced upon amino acid deprivation [32,51,56]. Although both regulators belong to the b-ZIP transcription factor family, they share no sequence homology with *S. pombe* Fil1, which is a GATA-type transcription factor [55]. Consistently, *S. pombe* lacks orthologs of both Gcn4 and Atf4. Thus, despite the conserved ability of *S. pombe*, *S. cerevisiae*, and mammals to induce the ISR in response to amino acid deprivation, the key regulators that are translationally modulated to launch a specific transcriptional program to promote cell survival are not conserved in terms of their functional domain composition. However, they are similar in the nature of the target genes they induce [55,57].

In this study, we found that the *hem1*Δ* hri1*Δ mutant strain exhibited poor levels of eIF2α phosphorylation under ALA deprivation compared to *hem1*Δ strains lacking either Hri2 or Gcn2. Under the same conditions, *hem1*Δ* hri1*Δ cells consistently showed higher levels of newly synthesized proteins than a *hem1*Δ strain expressing endogenous *hri1*^*+*^, or *hem1*Δ* hri1*Δ* hri2*Δ* gcn2*Δ cells expressing either *hri1*^*+*^ or *hri1*^*+*^*-GFP* alleles. However, after 19 h in ALA-free medium, all strains lacking Hri1 (*hri1*Δ) failed to resume growth upon the addition of exogenous hemin. These findings indicate that eIF2α phosphorylation by Hri1 is essential for cellular adaptation to ALA deprivation, as the absence of Hri1 prevents cells from re-establishing physiological homeostasis upon a re-exposure to hemin. To our knowledge, this is the first report of a fungal eIF2α kinase being activated in response to a stress directly linked to a defect in heme homeostasis. The phenotype associated with Hri1 loss aligns with our biochemical data, which demonstrate that Hri1 is exquisitely sensitive to hemin, binding to it with a *K*_*D*_ value of 0.11 × 10^-6^ M. This exquisite high affinity for hemin establishes Hri1 as a key sensor, capable of detecting submicromolar hemin concentrations. Interestingly, the hemin-binding affinity of Hri1 is one order of magnitude higher than that of Hri2 (*K*_*D*_ of 1.05 × 10^-6^ M), supporting its role as the primary sensor of the level of heme availability. This function enables Hri1 to coordinate the ISR when hemin levels become insufficient for the proper function of heme-dependent enzymes. Under severe heme depletion, the absence of heme binding to Hri1 promotes its activation, leading to eIF2α phosphorylation. In contrast, disruption of *hri1*^*+*^ (*hri1*Δ) impairs eIF2α phosphorylation under heme-depleted conditions. Among the *S. pombe* eIF2α kinases, Hri1 remains the least studied. Previous research has shown that Hri1 activation is associated with the transition from exponential growth to the stationary phase, specifically when cells reach an optical density of 4 units at 595 nm [34]. Additionally, Hri1 contributes to eIF2α phosphorylation when cells are transferred to a nitrogen-free medium [34]. Beyond these findings, no other stress conditions have been reported to activate Hri1, whereas Hri2 is known to respond to high temperatures, toxic metals, and glucose starvation [31,33–35]. Meanwhile, Gcn2 is primarily activated by H_2_O_2_, MMS, 3-AT, high salt, and nutrient limitation [33–35]. Although Hri1 and Hri2, and to a lesser extent Gcn2, share sequence and structural similarities within their predicted catalytic domains, they likely possess distinct regulatory domains that enable them to respond to specific cellular stresses.

Our results showed that the amino-terminal region of Hri1 is required for its ability to bind heme. Specifically, the deletion of the first 185 amino acids of Hri1 leads to a drastic reduction in its heme-binding capacity. Intriguingly, this amino-terminal portion of Hri1 lacks the two His residues (His^119^ and His^120^) that are critical for heme binding in the murine HRI protein and are conserved in human and rabbit HRI proteins [58]. Additionally, the kinase domain of *S. pombe* Hri1 lacks the Cys^409^Pro^410^ motif when compared to its murine, human and rabbit counterparts. In mice, this Cys^409^Pro^410^ motif has been identified as necessary for coordinating heme in conjunction with His^119^ and His^120^ residues [58]. Notably, *S. pombe* Hri1 is not the only heme-responsive eIF2α kinase missing these residues. HRI orthologs from chicken, Xenopus, and zebrafish retain only one of the two His residues and lack the CP motif or the Cys residue. Based on these observations, identifying the specific residues that constitute the heme-binding motif within the amino-terminal region and other unique regions of Hri1 will require further comprehensive dissection of the protein. It is intriguing that *S. pombe* possesses two HRI-related eIF2α kinases, Hri1 and Hri2, in addition to Gcn2. This suggests that *S. pombe* may have evolved an integrated stress response with distinct regulatory proteins to optimize adaptation to a broader range of stress conditions.

## Materials and methods

### Yeast strains and culture conditions

The genotypes of *S. pombe* strains used in this study are described in Table 1. All strains are derived from the parental strain FY435, which lacks the *hem1*^*+*^ gene (hem1∆) (TMY1) and is auxotrophic for ALA [40]. Under nonselective conditions, strains were grown on yeast extract plus supplements (YES) medium [59] with the indicated concentrations of ALA. For plasmid transformation by electroporation, strains were cultured on synthetic Edinburgh minimal medium (EMM), supplemented with ALA but lacking specific amino acids as required for plasmid selection. Deletion mutant strains were generated using a resistance gene disruption cassette engineered for repeated use in fission yeast [60]. This DNA cassette is composed of the kanamycin/G418 resistance marker (kanMX), flanked by loxP sequences on either side of the resistance gene. Each replacement cassette is bordered by short DNA regions homologous to the chromosomal sequences corresponding to the 5’- and 3’-untranslated regions of the target gene. Following gene replacement, the cassette was excised from the strain genome employing a Cre recombinase/loxP-mediated removal protocol [61].

**Table 1:** Yeast strains used in this study.

*S. pombe strain*	Genotype	Source
FY435	*h*^* +*^* his7–366 leu1–32 ura4-*∆*18 ade6-M210*	[37]
TMY1	*h*^* + *^*his7–366 leu1–32 ura4-*∆*18 ade6-M210 hem1*∆*::KAN*^*r*^	[37]
SPY046	*h*^* + *^*his7–366 leu1–32 ura4-*∆*18 ade6-M210 hem1*∆*::loxP hri1∆::KAN*^*r*^	This study
SPY047	*h*^* + *^*his7–366 leu1–32 ura4-*∆*18 ade6-M210 hem1*∆*::loxP hri2∆::KAN*^*r*^	This study
SPY048	*h*^* + *^*his7–366 leu1–32 ura4-*∆*18 ade6-M210 hem1∆::loxP gcn2∆::KAN*^*r*^	This study
ABY895	*h* ^* +*^ * his7–366 leu1–32 ura4-∆18 ade6-M210 hem1∆::loxP hri1∆::loxP hri2∆::loxP gcn2∆::KAN* ^*r*^	This study


For cell proliferation assays, precultures of the specified strains were grown in YES medium supplemented with ALA (25 µM). Once cultures reached the mid-logarithmic phase (OD_600_ of 0.5), cells were washed twice and diluted to an OD_600_ of 0.1 in YES medium either without ALA or with ALA supplementation (control). The cultures were then transferred to 96-well flat-bottom plates (Grenier) (200 µl per well) and incubated at 30^o^C in a SpectraMax M2 plate reader (Molecular Devices). Cell growth was monitored by measuring optical density at OD_600_ every 15 min for 19 h.

#### Plasmids.

A DNA fragment containing the *hri1*^*+*^ gene, spanning from -528 bp upstream of its initiator codon to +210 bp beyond its stop codon, was amplified by PCR using a primer set that introduced NotI and XmaI restriction sites at the ends of the PCR product. After purification and digestion, the PCR product was inserted into the corresponding sites of pJK148 [62], creating pJKhri1^+^. The *hri1*^*+*^ DNA fragment was also amplified without its stop codon and terminator region using a primer pair that introduced NotI and BamHI sites at the ends of the PCR product. The resulting NotI-BamHI PCR-amplified fragment was cloned into pJK148, yielding the plasmid pJKhri1^+^nostop. The GFP coding sequence was then amplified by PCR and inserted in-frame with the 3’-terminal coding sequence of *hri1*^*+*^ using the BamHI and SpeI sites found in pJKhri1^+^nostop. The resulting plasmid, pJKhri1^+^-GFP, was subsequently used to introduce a site-directed substitution, replacing the codon for Lys^253^ with a DNA triplet encoding alanine.

To create a plasmid containing the hygromycin (*HPH*) resistance gene, the pSP1 vector [63] was amplified in its entirety, except for the *LEU2* coding sequence, which was excluded from the PCR product. A second PCR reaction was performed to amplify the *HPH* resistance gene from the plasmid pFA6a-hphMX6 [64]. The two PCR products were then assembled using the Gibson Assembly method [65], resulting in the pSP1HPH plasmid, in which the *LEU2* nutritional cassette was replaced by the *HPH* resistance gene. To generate the plasmid pSPHS1-M7A, the HS1-M7A coding sequence, derived from p415GPDHS1-M7A [47], was isolated by PCR using primers designed to introduce XmaI and SacI restriction sites at the 5’ and 3’ ends of the amplified fragment. The XmaI-SacI DNA fragment was then inserted into the corresponding sites of pSP1HPH, producing a plasmid designated as pSPHS1-M7Anoprom. Next, the DNA region corresponding to the *tpx1*^*+*^ promoter was amplified by PCR from the plasmid pJK-1530tpx1^+^ [66]. This PCR product was subsequently inserted into pSPHS1-M7Anoprom at the ApaI and XmaI restriction sites, yielding the final plasmid pSPtpx1-HS1-M7A.

The coding sequences of *S. pombe hri1*^*+*^ and *hri2*^*+*^ were amplified by PCR using primers designed to introduce EcoRI and BamHI restriction sites at both ends of the PCR products. For Hri1ΔN, a similar approach was used, except that the first 185 codons, which encompass the putative N-terminal heme-binding domain of Hri1, were excluded. This resulted in a PCR product containing the *hri1*^*+*^ coding region corresponding to amino acids 186–704. All three purified PCR-amplified fragments were digested with EcoRI and BamHI and subsequently inserted into the corresponding sites of the plasmid pMAL-c2x (New England BioLabs, Beverly, MA).

#### Total heme measurements.

*hem1*Δ cells were precultured in the presence of 25 µM ALA, then washed, diluted and seeded to an OD_600_ of 0.1. One half of the cultures was incubated without ALA, while the other half was treated with ALA (100 µM) for 19 h. Following this treatment, the cultures were harvested, washed, resuspended in oxalic acid (20 mM), and kept at 4^o^C in the dark for 16 h, as previously described [44,67]. After the incubation period, an equal volume (500 µl) of oxalic acid (2 M) was added to each sample. The sample suspensions were then split, with one half incubated at 95^o^C for 30 min, whereas the other half was maintained at 25^o^C for the same duration. All samples were centrifuged for 2 min at maximum speed in a tabletop microcentrifuge, and porphyrin fluorescence (excitation: 400 nm; emission: 620 nm) was measured using a fluorometric assay, as previously described [44,67]. Additionally, heme concentrations were calculated from a standard curve generated using hemin chloride stock solutions, following the methodology described in previous studies [44–46,67].

#### Fluorescence microscopy.

*hem1∆* cells expressing the heme sensor HS1-M7A cells underwent a transition from ALA-sufficient to ALA-deficient conditions. These cells were analyzed using fluorescence microscopy to assess changes in the eGFP/mKATE2 fluorescence ratio after 19 h of incubation. The observed increase in the eGFP (green) fluorescence signal reflected reduced heme binding to the sensor and lower labile heme levels. Fluorescence and differential interference contrast (Nomarski) images were captured using a Nikon Eclipse E800 epifluorescence microscope (Nikon, Melville, NY) equipped with a Hamamatsu ORCA-ER digital cooled camera (Hamamatsu, Bridgewater, NJ). Green and red fluorescence signals were visualized at 1000 × magnification using emission filters of 485–525 nm for eGFP and 585–625 nm for mKATE2.

For the detection of the subcellular localization of Hri1-GFP and Hri1K253A-GFP proteins, the same fluorescence microscopy method was applied, except that only the green fluorescence signal was examined. *hem1∆ hri1∆ hri2∆ gcn2∆* cells expressing these proteins were cultured either in ALA-depleted or ALA-supplemented medium. Representative cell fields shown in the images were obtained from a minimum of three independent experiments. Furthermore, each condition was assessed in at least 200 cells to determine protein localization.

#### Heme sensor fluorescence measurements.

The *hem1∆* strain expressing the heme sensor HS1-M7A or harboring an empty plasmid was precultured in the presence of ALA (25 µM). Following cell growth, cultures were washed and seeded in ALA-free medium at a density of 1 × 10^7^ cells/ml, either without ALA supplementation or with ALA supplementation. These cultures were transferred in triplicate in black 96-well flat-bottom plates (Grenier Fluorotrac). Fluorescence signals of eGFP and mKATE2 were measured using a SpectraMax M2 microplate reader with the following excitation/emission wavelength pairs: 485 nm/525 nm for eGFP, and 585 nm/625 nm for mKATE2. Measurements were taken at 15-min intervals over a 19-h period. Background fluorescence from *hem1∆* cells carrying an empty plasmid (instead of HS1-M7A) was recorded and subtracted from the eGFP and mKATE fluorescence values. The average fluorescence intensities of eGFP and mKATE were determined, and the eGFP/mKATE ratio was calculated as described previously [68]. Since the measured value represents the heme-dependent repression of eGFP fluorescence divided by the heme-independent and constitutive mKATE fluorescence, a low eGFP/mKATE2 fluorescence ratio indicates a high concentration of labile heme, whereas an elevated eGFP/mKATE2 fluorescence ratio reflects a low concentration of labile heme.

#### RNA isolation and mRNA expression analysis by RT-qPCR assays.

Total RNA was extracted from cell cultures using a hot phenol method, as previously described [69]. Reverse transcription, cDNA synthesis, and qPCR reactions were performed following established procedures, as previously described [70]. Each target transcript listed in Table 2 was analyzed in experiments conducted with three biological replicates, with each sample reaction performed in triplicate. Results were considered valid if the target-specific fluorescent signal exhibited a C_t_ value ≤ 37 cycles, accompanied by positive and negative control reactions yielding productive amplification and no amplification, respectively. Log_2_ fold changes in transcript levels in response to ALA availability were calculated using the ΔΔCt method, with normalization to the internal control *act1*^*+*^ or *atb2*^*+*^ transcript [71–73]. The following equation was used for calculations: ΔΔCt = [(Ct gene-Ct ref) in -ALA] versus [(Ct gene-Ct ref) in +ALA]. The positions of the amplified regions for each transcript, relative to the first nucleotide of the initiation codon, are listed in Table 2.

**Table 2:** Target genes monitored by RT-qPCR assays.

Gene name	Amplified Positions (from the first nucleotide of the initiator codon)	Source
act1+	+173 to +280	[67]
atb2+	+516 to +626	This study
ctt1+	+359 to +472	This study
gcn2+	+662 to +768	This study
hri1+	+447 to +554	This study
hri2+	+361 to +457	This study
hsp16+	+114 to +234	This study
hsp9+	+4 to +117	This study
srx1+	+8 to +125	This study
zym1+	+3 to +132	This study
fio1+	+594 to +694	This study
frp1+	+352 to +452	This study
shu1+	+343 to +429	This study
str3+	+585 to +690	This study
eng1+	+433 to +545	This study
rps101+	+261 to +353	This study
pho1+	+389 to +482	This study

#### RNA-Seq library preparation and processing.

Total RNA isolated from *hem1*Δ cells under ALA-starved or ALA-replete conditions was quantified spectrophotometrically and assessed for integrity using an Agilent 2100 Bioanalyzer (Agilent Technologies). Library preparation began with mRNA enrichment using the NEBNext Poly(A) Magnetic Isolation Module (New England BioLabs). cDNA synthesis was performed using the NEBNext RNA First Strand Synthesis Module, followed by the NEBNext Ultra Directional RNA Second Strand Synthesis Module. Subsequent steps were carried out using the NEBNext Ultra II DNA library Prep Kit for Illumina, in combination with appropriate adapters and primers. The resulting libraries were quantified, and their average fragment sizes were determined using a LabChip GX instrument (Perkin Elmer). After normalization, the libraries were processed on a HiSeq 4000 sequencing system for 2 × 100 cycles, following the manufacturer’s instructions. The sequencing run was managed using Illumina’s HCS HD 3.4.0.38 control software, with real-time analysis performed using RTA v.2.7.7. Demultiplexing and FASTQ file generation were carried out using the bcl2fastq v2.20 program.

#### Bioinformatics.

Quality control of sequencing reads was assessed using R package fastqcr v0.1.3 [74]. Reads were trimmed to remove adapters, low-quality 3’ ends, and sequences shorter than 20 nucleotides using Trim Galore v0.6.6. Paired-end reads from RNA-Seq were aligned to the *S. pombe* reference genome (ASM294v2.25) and annotated (GTF version ASM294v2.58) using the R package Rsubread v2.18.0 [74]. Differential transcript abundance analysis was performed using the R packages edgeR v4.2.2 and limma v3.60.6 [75]. This analysis identified statistically significant differentially expressed genes between cultures incubated with and without ALA supplementation. The resulting data table includes P values, adjusted P values (corrected using the Benjamini-Hochberg procedure [76], and log2 fold changes for each gene. Transcripts with a log_2_ fold change greater than 1.5 or less than -1.5 (with adjusted p-value < 0.05) were considered differentially enriched and used for further analysis. Gene ontology enrichment analysis of significantly upregulated and downregulated genes was performed using the AnGeLi tool [77] against a total of 5,677 transcripts considered in the RNA-Seq analysis. Enrichments with a p-value > 0.05 were considered significant.

#### Global translation assay.

The indicated strains were seeded at an OD_600_ of 0.1 in YES medium, with or without ALA supplementation. After 18 h and 30 min, cells were treated with 10 μM L-homoproparglyglycine (HPG) for 30 min (4 μl from a 50 mM stock solution in water per 20 ml culture). Following the treatment, cells were fixed with 70% ethanol for 15 minutes at 4˚C. Fixed cells were washed with PBS and permeabilized using 1% Triton X-100 in PBS for 30 min at 30˚C in the dark. Cells were then centrifuged, resuspended in PBS supplemented with BSA (1%), and incubated at 25˚C on a rocker for 60 min in the dark. After incubation, cells were centrifuged, resuspended in 1 × Click-iT reaction buffer, and transferred as aliquots into microcentrifuge tubes for HPG incorporation assays. Half of the aliquots were not mixed with Alexa Fluor 647 Azide, as they were used to substract background non-specific fluorescence from all readings. A typical Click-iT reaction mix contained 440 µl of 1 × Click-iT reaction buffer, 10 µl of CuSO_4_ (100 mM), 40 µl of 10 × Click-iT buffer additive, and 10 µl of Alexa Fluor 647 Azide (1 µM) (Thermo Fisher Scientific A10277), ensuring that Alexa Fluor 647 Azide was added last. When indicated, 0.1 mg/ml of cycloheximide (CHX) was added to the cultures 30 min prior to HPG addition to inhibit global cellular translation. Fluorescently labelled HPG with Alexa Fluor 647 was measured using a SpectraMax M2 microplate reader with an excitation/emission wavelength pair of 650/671 nm. Measurements were taken at the 19-h time point. Results are presented as averages ± SD of three independent experiments, each performed in biological triplicate.

#### Protein Extraction and immunoblot analysis.

To assess protein steady-state levels, whole-cell extracts were prepared by glass bead disruption using HEGN_100_ lysis buffer, which contained 20 mM 4-(2-hydroxyethyl)-1-piperazineethanesulfonic acid (HEPES), pH 7.9, 100 mM NaCl, 1 mM ethylenediaminetetraacetic acid (EDTA), 10% glycerol, 0.1 mM Na_3_VO_4_, 1 mM PMSF, 1 mM dithiothreitol (DTT), and a complete protease inhibitor mixture (Sigma-Aldrich; P8340). Cell lysis was conducted using a FastPrep-24 instrument (MP Biomedicals, Solon, OH). Equal concentrations of protein samples were resolved by electrophoresis on 8% sodium dodecyl sulfate (SDS)-polyacrylamide gels. After protein transfer from gel to membrane, phosphorylated and unphosphorylated forms of eIF2α were detected using the monoclonal anti-phospho-eIF2α antibody 119A11 and the polyclonal eIF2α antibody 9722, respectively (Cell Signaling Technology). For HS1-M7 and Hri1-GFP detection, the monoclonal anti-GFP antibody B-2 (Santa Cruz Biotechnology) was used, whereas α-tubulin was immunodetected using the monoclonal anti-α-tubulin antibody B-5-1-2 (Sigma-Aldrich). After incubation with the primary antibodies, membranes were washed and incubated with the appropriate horseradish peroxidase (HRP)-conjugated secondary antibodies (Amersham Biosciences). Protein signals were revealed using enhanced chemiluminescence (ECL) reagents (Amersham Biosciences) and visualized using an ImageQuant LAS 4000 instrument (GE Healthcare).

#### Heterologous protein expression in E. coli and absorbance spectroscopy.

Plasmids pMAL-hri1^+^, pMAL-Hri1ΔN, and pMAL-hri2^+^ were transformed into *E. coli* Rosetta (DE3)pLysS cells. Fresh transformants were grown in LB media to an OD_600_ of 0.5. At this stage, heterologous protein expression was induced by adding 0.2 mM IPTG, followed by incubation at 23^o^C for 20 h in the presence of 2% ethanol. Cells were then harvested and sonicated in Buffer A (50 mM Tris–HCl, pH 7.5, 150 mM NaCl, 10% sucrose, 50 μg/mL lysozyme, 1% Triton X-100, and 1x protease inhibitor cocktail (Sigma-Aldrich; P8340)). The supernatant fraction containing soluble proteins was incubated with a 2-ml suspension of amylose resin for 18 h at 4^o^C. After washing the resin beads, MBP-Hri1, MBP-Hri1Δ, or MBP-Hri2 proteins bound to the resin were eluted in the presence of Buffer A supplemented with 20 mM maltose. The eluted fractions containing heterologous proteins were dialyzed to remove maltose and either subjected to a second round of purification using the same affinity resin or directly analyzed for their heme-binding properties. Prior to heme-binding assays, 100 µg of purified proteins were dephosphorylated using 800 units of lambda protein phosphatase (New England BioLabs; P0753S) in 1 × metallophosphatase buffer (50 mM HEPES, pH 7.5, 100 mM NaCl, 2 mM DTT, and 0.01% Brij 35) supplemented with 1 mM MnCl_2_. The reaction was carried out at 30^o^C for 30 min, as previously described [78].

A stock solution of hemin (3 mM) was freshly prepared in 0.1 M sodium hydroxide (NaOH) as previously described [79]. To assess the interaction of hemin with Hri1, Hri1ΔN, or Hri2, increasing concentrations of the indicated purified proteins were added to hemin (5 μM) in buffer H (20 mM HEPES, pH 7.4, and 40% dimethyl sulfoxide). Absorbance spectra were recorded in the 350–600 nm range using a BioMate spectrophotometer. The formation of the protein-hemin complex was monitored by measuring decreases in absorbance at the Soret peak. Graphs were generated to illustrate changes in absorbance at the Soret peak as a function of protein concentrations. Data analysis was performed using GraphPad Prism software (version 10.4.1).

## Supporting information

S1 Appendix Correlation between RNA-seq replicates and validation of RNA-seq transcript abundance changes by RT-qPCR. Related to Fig 2.
(PDF)

S1 Data The raw data shown are the values used to generate the graphs in Fig 1, panels B, C and G. (XLSX)

S2 Data The raw data shown are the values used to generate the graphs, heat maps, and clusters in Fig 2, panels B, C, D, E, F, G and H. (XLSX)

S3 Data The raw data shown are the values used to generate the graphs in Fig 3, panels C and E. (XLSX)

S4 Data The raw data shown are the values used to generate the graphs in Fig 4, panels A, B and D. (XLSX)

S5 Data The raw data shown are the values used to generate the graphs in Fig 5, panels A and E. (XLSX)

S6 Data The raw data shown are the values used to generate the graphs in Fig 6, panels A, B and C. (XLSX)

S1 Table Total of 579 genes exhibited high expression levels in ALA-starved *hem1*Δ cells. Related to Fig 2.
(XLSX)

S2 Table Total of 170 genes exhibited decreased expression levels in ALA-starved *hem1*Δ cells. Related to Fig 2.
(XLSX)
