# Supplementary material for: The heme-regulated inhibitor kinase Hri1 is activated in response to aminolevulinic acid deficiency in Schizosaccharomyces pombe
Source: PLoS Genet. 2025 Jul 16;21(7):e1011797. doi: 10.1371/journal.pgen.1011797 (PMC12303383; doi:10.1371/journal.pgen.1011797)
Supplement: S1 Appendix — Related to Fig 2. (PDF) [file pgen.1011797.s001.pdf]

Supplementary Information

The heme-regulated inhibitor kinase Hri1 is activated in response to aminolevulinic acid deficiency in *Schizosaccharomyces pombe*.

Samuel Plante, Ariane Brault, Mariano Avino, Hajer Sakouhi, Florie Lo Ying Ping, Tobias Vahsen, and Simon Labbé*.

Figure S1: Correlation between RNA-seq replicates and validation of RNA-seq transcript abundance changes by RT-qPCR. Related to Figure 2.

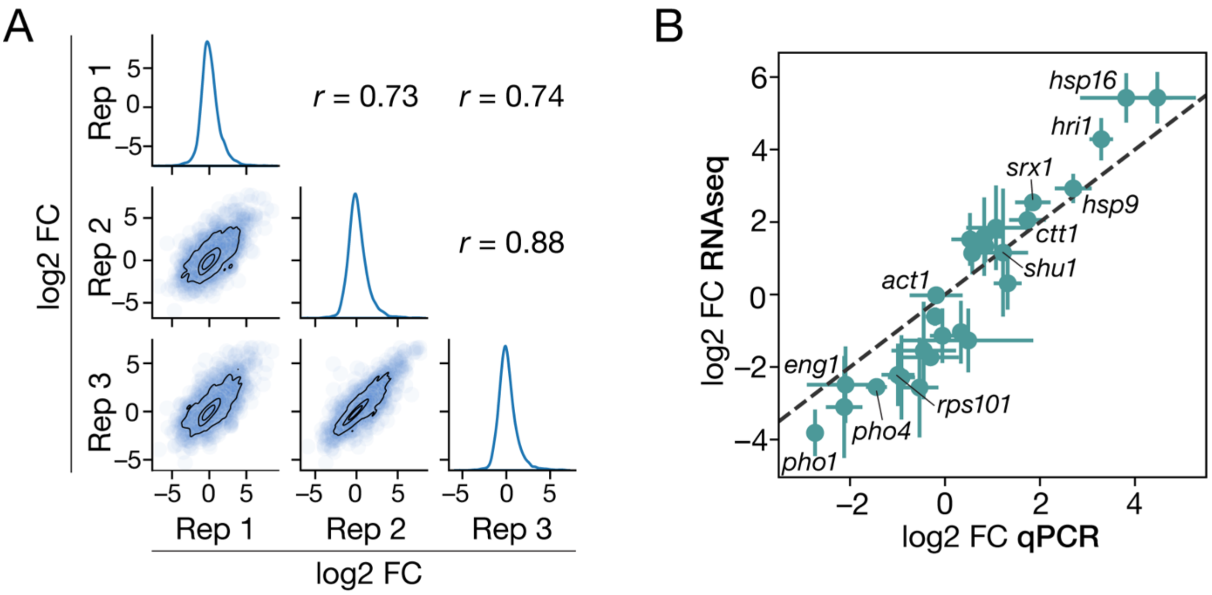

Figure S1 - related to Figure 2
Plante et al.

Figure S1. Correlation between RNA-seq replicates and validation of RNA-seq transcript abundance changes by RT-qPCR. Related to Figure 2.

A) Correlation matrix between replicates. Lower corner shows scatter plots and respective density plots of log2FC values of the indicated replicate. The diagonal shows the distribution of log2FC values in each replicate. The upper corner shows the Pearson' r coefficient between the indicated log2FC replicate.

B) Scatterplot of log2FC values from RNA-seq experiments for a subset of genes, and the respective log2FC value obtained by RT-qPCR on independent biological replicated. Plotted are mean values ± S.D from 3 independent replicates. The dashed diagonal at y=x illustrates a significant correlation between methods.
